# Tampon use, environmental chemicals and oxidative stress in the BioCycle study

**DOI:** 10.1186/s12940-019-0452-z

**Published:** 2019-02-11

**Authors:** Jessica Singh, Sunni L. Mumford, Anna Z. Pollack, Enrique F. Schisterman, Marc G. Weisskopf, Ana Navas-Acien, Marianthi-Anna Kioumourtzoglou

**Affiliations:** 1 Department of Environmental Health Sciences, Columbia University Mailman School of Public Health, New York, NY USA; 2 Eunice Kennedy Shriver National Institute of Child Health and Human Development, Epidemiology Branch, Bethesda, MD USA; 3 Department of Global and Community Health, College of Health and Human Services, George Mason University, Fairfax, Virginia USA; 4 Department of Environmental Health, Harvard TH Chan School of Public Health, Boston, MA USA

**Keywords:** Tampons, BioCycle, Metals, Oxidative stress, Menstrual cycle

## Abstract

**Background:**

Tampons are used by up to 86% of US women and are a rarely considered potential source of pesticide and metal exposure. Tampons may be of particular concern given the likely higher absorption that occurs in the vagina. Our objective was to examine the potential associations between tampon use and metal concentrations, and biomarkers of inflammation and oxidative stress among healthy women.

**Methods:**

We used information from a prospective cohort of 259 regularly menstruating women, aged 18–44, followed for two menstrual cycles. Tampon use was assessed using information provided in participant study diaries. Metal concentrations were measured from a blood sample collected at enrollment. Oxidative stress and inflammation biomarker concentrations were determined from blood samples collected at up to 8 clinic visits for each cycle. Linear regression models were used to estimate associations of tampon use with metal exposure, and linear mixed models to estimate associations of tampon use with inflammation and oxidative stress biomarkers at different times during the menstrual cycle.

**Results:**

We observed non-significantly higher mean levels of mercury for tampon users compared to non-tampon users (exp(β) = 1.25, 95% CI = 0.93, 1.68). We found no evidence of an association between tampon use and inflammation biomarkers. We observed consistently higher isoprostane levels, an oxidative stress biomarker, among tampon users compared to non-tampon users (e.g. exp.(β) = 1.05, 95%CI = 0.96, 1.16, for the average isoprostane during the menstruating week); however, these results were not statistically significant.

**Conclusions:**

While our results are not statistically significant, we observed suggestive associations between tampon use and elevated levels of mercury and oxidative stress biomarkers. Although our finding should be interpreted in light of our limitations, they indicate that tampons may be a source of exposure to metals and chemicals that have been largely ignored, and any related health effects are an important public health concern.

**Electronic supplementary material:**

The online version of this article (10.1186/s12940-019-0452-z) contains supplementary material, which is available to authorized users.

## Background

The vaginal route is a potentially important yet understudied route of chemical exposure. Feminine hygiene products are personal care products used by many women for menstruation and vaginal discharge. Tampons, a commonly used feminine hygiene product, with 50–86% of women in the United States report using them during menstruation, are inserted into the vagina to absorb menstrual blood [1]. Notably, the vagina is an effective delivery route of drugs to the systemic circulation system [2], suggesting that it could also effectively deliver other compounds, like toxic chemicals, to the circulation. This is due to the abundance of arteries, blood and lymphatic vessels in the walls of the vagina mucosa, and the fact that absorption through this route bypasses first-pass metabolism, by directly entering the peripheral circulation [2]. For instance, vaginal administration of estradiol results in significantly higher blood serum levels compared to oral administration [3]. Furthermore, vulvar and vaginal tissues are more hydrated and permeable compared to the skin on the rest of the body, which may make these more susceptible to chemical exposure [4, 5]. Moreover, in addition to systemic exposure, vaginal exposure to chemicals and drugs can also have local effects on vaginal and cervical tissue [6]. Therefore, if tampons do contain harmful chemicals, tampon use may [1] be a potentially important source of these chemicals via the vaginal route given the rapid absorption that occurs in the vagina and the cumulative exposure to tampons over a women’s reproductive life and [2] may also affect the epithelial integrity of vaginal and cervical cells, potentially increasing susceptibility to sexually transmitted infections.

Tampons are a potential source of chemical exposure. Most tampons are made of cotton or cotton blends, although some tampons are made solely of rayon. Agricultural soils contain both metals and pesticides, which originate from the use of fertilizers, sewage sludge, irrigation water and fertilizers containing metals, and the direct application of pesticides [7, 8]. The accumulation of metals in soils of cotton fields is an emerging public health concern because of rapid industrial development and increasing reliance on agrochemicals [7]. Studies have shown that cotton plants can bio-accumulate various metals, including lead, copper, zinc, and cadmium in different parts of the plant [9, 10]. The chlorine bleaching process may contribute to dioxins and furans in tampons and fragrance chemicals, such as phthalates, are likely to be found in scented products [1, 11]. Identifying specific chemicals is challenging due to the lack of labeling requirement for tampons.

Metals like cadmium, lead, and mercury are known environmental toxicants [12], and are thus especially of concern. Metals have oxidation-reduction properties that have been associated with enhancing oxidative damage, an important risk factor in the development of chronic inflammatory disease [12, 13]. Cadmium exposure has been linked to kidney and cardiovascular disease in humans [12, 13]. Lead and mercury exposures have been associated with negative impacts on the nervous system and with cardiovascular disease [12, 14]. Pesticides like organophosphates are also known to induce oxidative stress, and are related to multiple adverse health effects [15, 16].

Therefore, women who use tampons may be exposed to metals and/or pesticides if the cotton was grown in metal contaminated soils or if pesticides were applied to the cotton used in the tampons. The bleaching process can create dioxins to which women can then be exposed [17], and fragrance chemical exposure is plausible though understudied. If women are exposed to metals and/or pesticides via this source then they may be vulnerable to multiple adverse health effects. However, since studies thus far have only investigated oral, dermal or inhalation exposures, it is unknown if vaginal exposure will have comparable findings.

Tampons are, thus, a not yet assessed potential source of exposure to various metals and pesticides, which should be of concern because of the multiple health effects related to the exposure of these environmental chemicals. To our knowledge, there are no peer-reviewed studies assessing the relationship between tampon use and metal or pesticide concentrations. For this study, we used existing data from the BioCycle Study to assess whether tampon use is related to increased metal concentrations in blood. As pesticides are not available in BioCycle, this hypothesis could not be directly tested in this study. However, we hypothesized that any potential exposure to metals and pesticides through tampon use can be related to increased inflammation and oxidative stress. We thus assessed the association between tampon use and blood biomarker levels for inflammation and oxidative stress.

## Methods

### Study population

The BioCycle Study is a prospective cohort study that was designed to understand the relationship between reproductive hormone levels and oxidative stress during the menstrual cycle [18]. Between 2005 and 2007, 259 healthy, regularly menstruating women – who self-reported menstrual cycle lengths between 21 and 35 days for the past 6 months – were enrolled from Western New York for up to two menstrual cycles [18]. Exclusion criteria included women who planned to or were actively trying to conceive, a self-reported body mass index (BMI) < 18 or > 35 kg/m^2^ at baseline, not between the ages of 18 and 44 years, and histories of gynecologic or other chronic diseases [18]. Of the 259 women, 9 were followed for one menstrual cycle, and 250 were followed for two menstrual cycles [18]. Blood samples were collected at clinic visits for each cycle corresponding to specific phases of the menstrual cycle, including menstruation, mid- and late follicular phase, luteinizing hormone surge, estimated day of ovulation, and early-, mid-, and late luteal phase. Fertility monitors (Clearblue Easy Fertility Monitor; Inverness Medical, Waltham, MA) and personal cycle length histories were used to determine the timing of visits [19]. Further information on study design can be found elsewhere [18].

### Exposure assessment

At the time of enrollment, participants were asked to complete a health questionnaire, which included questions related to their menstrual cycle. Participants were asked to report the type of feminine hygiene product they used while menstruating (sanitary napkins, tampons, both, or other). Participants were also asked to complete a daily diary during the study which asked questions regarding their specific feminine hygiene product use if they reported any menstrual bleeding during their participation in the study [20].

To assess tampon use, we considered any use during the study period (any use versus none) using the information provided in the diaries. We also calculated the number of tampons used per cycle and used this variable in sensitivity analyses.

### Outcome assessment

#### Metals

Cadmium, lead, and mercury were measured from a single whole-blood sample that was collected at enrollment, an average of 16 days before the first clinic visit, in ethylenediaminetetraacetic acid (EDTA) purple-topped tubes, which were prescreened for trace metals [21]. Blood cadmium, lead and mercury levels were determined by inductively coupled plasma mass spectrometry at the CDC’s Division of Laboratory Sciences, National Center for Environmental Health [21]. Mercury levels represent the total mercury concentration in blood from all forms of mercury [21]. The limits of detection (LODs) for cadmium, lead and mercury were 0.20 μg/L (27% < LOD), 0.25 μg/dL (0% < LOD) and 0.33 μg/L (12% < LOD), respectively. All machine-read values were included in analyses, even those that were below the LOD.

#### Oxidative stress and inflammation biomarkers

Oxidative stress and inflammation biomarker concentrations were determined from blood samples collected at each of the 8 clinic visits for both cycles [22–25]. F2-8α isoprostanes (isoprostane), thiobarbituric acid reactive substances (TBARS), human serum paraoxonase 1 arylesterase (PON1A), and human serum paraoxonase 1 paraoxonase (PON1P) are biomarkers of oxidative stress, and C-reactive protein (CRP) is a biomarker of chronic inflammation. Isoprostane and TBARS levels were measured in coagulated plasma collected in EDTA purple-topped tubes, and PON1A, PON1P, and CRP levels were measured in anticoagulated serum collected in red-topped tubes. Isoprostane levels were determined using a gas chromatography-mass spectrometry-based method at the Molecular Epidemiology and Biomarker Research Laboratory of the University of Minnesota (Minneapolis, MN) [24]. TBARS levels were determined using OxiTech reagent kits at the Oxidative Stress Research Laboratory of the University at Buffalo [22, 24]. PON1A and PON1P levels were determined using the Cobas Fara II chemistry analyzer [25]. CRP levels were determined using IMMULITE 2000 High Sensitivity CRP chemiluminescent immunoassay [23].

To assess the short and long-term effects of tampon use on oxidative stress and inflammation, i.e. both during and post-menstruation, five variables representing different time periods in the women’s menstrual cycle were created. Specifically, we assessed biomarker levels during menses and the early-follicular phase visits separately, menstruating week (by estimating the average of the menses and early-follicular phase visits), the average biomarker concentration during entire cycle, and, finally, the average concentrations during the cycle post-menstruation (the average of visits around expected ovulation and through the luteal phase). For CRP, values larger than 10 mg/L were excluded from analysis, as those values are indicative of infection [26].

### Statistical analysis

Descriptive statistics were calculated for demographic characteristics, metals, and oxidative stress and inflammation biomarkers by tampon use during the study. We calculated the geometric means and standard deviations for the metals, oxidative stress and inflammation biomarkers (using the cycle-wide averages) by tampon use. Since these associations have never been investigated before, to identify potential confounders we conducted univariate analyses to assess the relationship between potential confounders and the dependent variables (metals and biomarker concentrations), and the independent variable (tampon use).

We employed linear regression models to estimate the association between tampon use and metal exposure. Although metals were measured at baseline, and thus before the reported tampon use, we assume that tampon use patterns within woman are consistent over time and reflective of past cycles as well. This is also evident in our data, as the estimated intraclass correlation coefficient (ICC) for tampon use was 0.83. Because the metal concentrations were highly skewed, these were natural log-transformed to meet the assumption of normally distributed residuals of the linear regression. We used three main modeling approaches, starting from a crude model (no covariates other than tampon use included; Model 1), a basic model including age, BMI, smoking, education, race, parity, and physical activity (Model 2), and an extended model additionally adjusting for history of birth control use and marital status (Model 3). Information on all potential confounders was obtained from the baseline questionnaires. Additionally, models for mercury exposure were adjusted for fish consumption because exposure might reflect increased fish and seafood consumption [27], and could be related to tampon use through socioeconomic status (SES). For this analysis, fish consumption was assessed as the sum (continuous) of four variables from a food frequency questionnaire (FFQ) administered at baseline: 1) fried fish and shellfish and fish sandwich, 2) shellfish (not fried), 3) white fish such as sole, halibut, snapper and cod, 4) and dark fish such as salmon, mackerel and bluefish. For mercury, 5 participants were excluded from the models because they had mercury concentrations equal to 0 μg/L, and thus the natural log was not defined. Of these, 1 participant did not have information about tampon use, 2 were tampon users and 2 were non-tampon users. Therefore, exclusion of these participants from analyses is likely not informative and would not induce bias. As a sensitivity analysis, and to include all participants, we also used the following transformation for mercury: log(Hg) + 1 and repeated our analysis.

We employed linear mixed regression models to estimate the association between tampon use and inflammation and oxidative stress biomarker concentrations, using random intercepts for each subject to account for within subject clustering, in separate models for each menstrual cycle time period. Because the biomarker concentrations were highly skewed, these were natural log-transformed to meet the assumption of normally distributed residuals of the linear regression. The same three levels for confounding adjustment described above were used.

For both the metals and biomarkers we repeated analyses restricting to non-smokers. Finally, we also repeated Model 2 for the biomarkers including the number of tampons used per cycle (continuous) as the exposure of interest to evaluate a potential dose-response relationship. We first included the number of tampon used as a linear term in the model. To assess the assumption of linearity, we repeated analyses using generalized additive mixed models (GAMMs) and a penalized spline for the number of tampons used. The number of degrees of freedom for the spline was selected using the generalized cross-validation criterion (GCV).

We present the results as the ratio of the expected geometric mean for those who used tampons over those who did not (exp(β)) and 95% confidence intervals (95% CI). All statistical analyses were conducted using the R Statistical Software, version 3.4.0 (Foundation for Statistical Computing, Vienna, Austria).

## Results

### Metals and oxidative stress and inflammation biomarkers in blood

A total of 158 (62%) BioCycle participants were tampon users (Table 1). Among them, a median of 4 tampons were used per cycle (IQR: 3–5). Tampon users were more likely to be white (79.6%), have more than a high school education (89.5%), be single (68.4%), non-smokers (94.7%), used birth control in the past (62.0%), nulliparous (68.5%) and be highly physical active (64.5%). For tampon users, the geometric mean cadmium level was 0.26 μg/L, lead level was 0.85 μg/L, and mercury level was 1.08 μg/L. The geometric mean PON1A level was 113.12 μmol/min/L, isoprostane level was 47.80 pg/mL, PON1P level was 179.35 μmol/min/L, and CRP level was 1.32 mg/L. TBARS blood levels were similar for both tampon and non-tampon users.

**Fig. 1:**
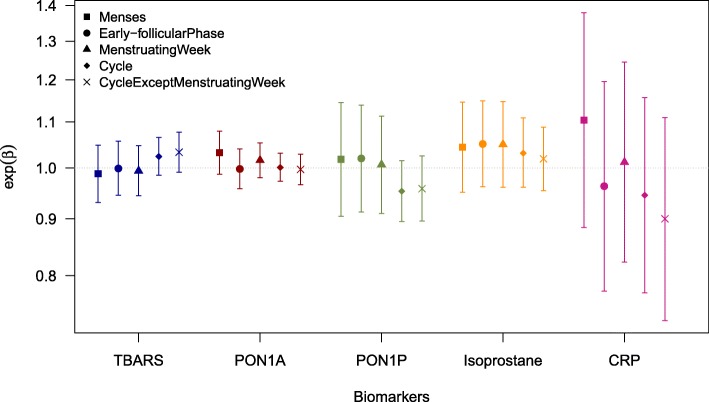
Ratio of the expected geometric mean for those who used tampons over those who did not (exp(β)) and 95% confidence intervals for Blood Oxidative Stress and Inflammation Biomarkers for Model 2

**Table 1:** Demographics and Metal and Biomarker concentrations by Tampon Use

	Tampon Users (*n* = 158)	Non-Tampon Users (*n* = 97)
Demographics		
Race/ethnicity (%)		
White	79.	26.88
Black	8.55	39.78
Other	11.84	33.33
Education (%)		
< High school	10.53	17.20
> High school	89.47	82.80
Marital Status (%)		
Ever Married	31.58	18.28
Single	68.42	81.72
Smoking status (%)		
Yes	5.26	1.08
No	94.74	98.92
Birth Control Use (%)		
Yes	62.00	40.86
No	38.00	59.14
Parous (%)	31.54	20.65
Nulliparous (%)	68.46	79.35
Physical Activity (%)		
Low	5.26	13.98
Medium	30.26	46.24
High	64.47	39.78
Age [years (mean (SD))]	27.87 (8.66)	27.16 (7.73)
BMI [(kg/m^2^ (mean (SD))]	23.91 (3.63)	24.43 (4.27)
Metal Concentrations		
Cadmium [μg/L (GM (SD))]^a^	0.26 (1.90)	0.33 (1.90)
Lead [μg/dL (GM (SD))]^a^	0.85 (1.53)	1.01 (1.62)
Mercury [μg/L (GM (SD))]^a^	1.08 (2.75)	1.01 (2.47)
Biomarker Concentrations		
TBARS [nmol/mL (GM (SD))]^b^	0.85 (1.27)	0.85 (1.25)
PON1A [μmol/min/L (GM (SD))]^b^	113.12 (1.24)	111.39 (1.22)
PON1P [μmol/min/L (GM (SD))]^b^	179.35 (1.84)	212.90 (1.83)
Isoprostane [pg/mL (GM (SD))]^b^	47.80 (1.37)	46.11 (1.45)
CRP [mg/L (GM (SD))]^b^	1.32 (6.48)	3.17 (13.60)

^a^Geometric mean (standard deviation) blood metal levels

^b^Geometric mean biomarker concentrations (standard deviation) measured from the created cycle variable

### Metals, oxidative stress and inflammation biomarkers by tampon use

Results for models 1, 2, and 3 for blood metals levels were overall similar (Table 2). After adjustment for age, ethnicity, education, smoking status, BMI, parity, physical activity, and fish consumption (for mercury exposure), tampon users had non-significantly higher geometric mean ratio of blood mercury compared to non-users (exp(β) = 1.25, 95% CI = 0.93, 1.68) (Table 2, Model 2). When using the log(Hg) + 1 transformation for mercury, the results were similar and statistically significant (exp(β) = 1.16, 95% CI = 1.02, 1.29).

**Table 2:** exp(β)^a^ of Tampon Use and 95% Confidence Intervals (95% CIs) for Blood Metals

	Model 1^b^	Model 2^c^	Model 3^d^
	exp(β) (95% CI)	exp(β) (95% CI)	exp(β) (95% CI)
Metals			
Cadmium (μg/L)	0.81 (0.69, 0.96)	0.94 (0.78, 1.12)	0.94 (0.78, 1.14)
Lead (μg/dL)	0.85 (0.75, 0.95)	0.92 (0.80, 1.05)	0.91 (0.80, 1.05)
Mercury (μg/L)^e^	1.07 (0.83, 1.37)	1.25 (0.93, 1.68)	1.24 (0.92, 1.67)

^a^Ratio of the expected geometric mean for those who used tampons over those who did not

^b^Crude model (no covariates other than tampon use included)

^c^Basic model including age, BMI, smoking, education, race, parity, and physical activity

^d^Extended model additionally adjusting for birth control use and marital status

^e^Models 2 and 3 for mercury additionally adjusted for fish consumption

Results for the biomarker models are presented in Table 3 and Figure 1. After adjustment, tampon users had higher mean levels of blood isoprostane during menses and the early-follicular phase, and menstruating week compared to non-tampon users (Table 3, Model 2). For TBARS, tampon users had non-significantly higher levels for the entire cycle, and the cycle except the menstruation week. For PON1P, tampon users had non-significantly lower levels for the cycle and the cycle except the menstruation week. For CRP, the results were mixed with wide confidence intervals across the different time periods in the women’s menstrual cycle.

**Table 3:** exp(β)^a^ of Tampon Use and 95% Confidence Intervals (95% CIs) for Blood Oxidative Stress and Inflammation Biomarkers

	Model 1^b^	Model 2^c^	Model 3^d^
	exp(β) (95% CI)	exp(β) (95% CI)	exp(β) (95% CI)
TBARS (nmol/mL)			
Menses	0.98 (0.93, 1.03)	0.99 (0.93, 1.05)	0.99 (0.94, 1.05)
Early-follicular phase	0.99 (0.94, 1.04)	1.00 (0.95, 1.06)	1.01 (0.95, 1.06)
Menstruating Week	0.99 (0.94, 1.03)	0.99 (0.94, 1.05)	1.00 (0.95, 1.05)
Cycle	0.99 (0.94, 1.03)	1.02 (0.99, 1.07)	1.00 (0.95, 1.05)
Cycle except menstruating week	1.02 (0.98, 1.06)	1.03 (0.99, 1.08)	1.04 (0.99, 1.08)
PON1A (μmol/min/L)			
Menses	1.03 (0.99, 1.07)	1.03 (0.99, 1.08)	1.03 (0.99, 1.08)
Early-follicular phase	1.00 (0.96, 1.04)	1.03 (0.99, 1.08)	1.00 (0.96, 1.04)
Menstruating Week	1.01 (0.98, 1.05)	1.02 (0.98, 1.05)	1.00 (0.96, 1.04)
Cycle	1.00 (0.97, 1.03)	1.00 (0.97, 1.03)	1.00 (0.97, 1.03)
Cycle except menstruating week	1.00 (0.97, 1.03)	1.00 (0.97, 1.03)	1.00 (0.97, 1.03)
PON1P (μmol/min/L)			
Menses	0.87 (0.78, 0.98)	1.02 (0.91, 1.15)	1.02 (0.91, 1.15)
Early-follicular phase	0.90 (0.81, 1.00)	1.02 (0.91, 1.14)	1.02 (0.91, 1.14)
Menstruating Week	0.90 (0.82, 0.99)	1.01 (0.91, 1.11)	1.01 (0.91, 1.12)
Cycle	0.91 (0.86, 0.97)	0.95 (0.90, 1.02)	0.95 (0.89, 1.02)
Cycle except menstruating week	0.91 (0.86, 0.98)	0.96 (0.90, 1.03)	0.96 (0.90, 1.03)
Isoprostane (pg/mL)			
Menses	1.09 (1.00, 1.19)	1.04 (0.95, 1.15)	1.04 (0.95, 1.14)
Early-follicular phase	1.09 (1.01, 1.19)	1.05 (0.96, 1.15)	1.05 (0.96, 1.15)
Menstruating Week	1.09 (1.01, 1.19)	1.05 (0.96, 1.15)	1.05 (0.96, 1.14)
Cycle	1.07 (1.00, 1.15)	1.03 (0.96, 1.11)	1.03 (0.96, 1.11)
Cycle except menstruating week	1.06 (0.99, 1.12)	1.02 (0.95, 1.09)	1.02 (0.95, 1.09)
CRP (mg/L)			
Menses	1.13 (0.91, 1.41)	1.10 (0.88, 1.38)	1.06 (0.85, 1.32)
Early-follicular phase	0.93 (0.75, 1.15)	0.96 (0.78, 1.20)	0.93 (0.75, 1.16)
Menstruating Week	0.99 (0.81, 1.22)	1.01 (0.82, 1.25)	0.98 (0.80, 1.20)
Cycle	0.94 (0.77, 1.12)	0.95 (0.77, 1.16)	0.94 (0.76, 1.15)
Cycle except menstruating week	0.89 (0.73, 1.10)	0.90 (0.73, 1.11)	0.90 (0.73, 1.11)

^a^Ratio of the expected geometric mean for those who used tampons over those who did not

^b^Crude model (no covariates other than tampon use included)

^c^Basic model including age, BMI, smoking, education, race, parity, and physical activity

^d^Extended model additionally adjusting for birth control use and marital status

When restricting analysis to non-smokers, the overall results were similar to the results presented in Table 3. For TBARS, tampon users had higher levels for the entire cycle (Additional file 1: Table S1). Analysis of the continuous variable (number of tampons used per cycle) showed similar results to the binary tampon use analysis (Additional file 1: Table S2), with no evidence for deviations from linearity, as selected by GCV in GAMM models (estimated df = 1).

## Discussion

We observed increased, albeit not significantly so, levels of blood mercury, and decreased levels of blood cadmium and lead for women who used tampons, although this decrease was much weaker than the increase for mercury. We also observed increased levels of oxidative stress biomarkers during different times of the menstrual cycle, with isoprostane having increased levels during the menstruation week for women who used tampons. Moreover, women who used tampons tended to have higher TBARS and lower PON1P levels during the cycle except from the menstruation week. However, our results were not statistically significant. Therefore, our study provides some suggestive evidence that there may be an association between tampon use and oxidative stress, but more studies are warranted.

Metals have oxidation-reduction properties that have been associated with enhancing oxidative damage, which is an important risk factor in the development of chronic inflammatory disease [12, 13]. Metal exposure may depress the function of lipid-associated enzymes, which are thought to protect against lipid peroxidation and may have implications for cardiovascular disease risk with aging and cumulative exposures [25]. Pesticides are also known to induce oxidative stress and inflammation, and are related to multiple adverse health effects [16, 28]. For example, exposure to organophosphates can induce oxidative stress in humans, showing evidence of lipid peroxidation in human erythrocytes [15]. In our study, we found isoprostane and TBARS, biomarkers of lipid peroxidation, to be non-significantly higher in tampon users than non-tampon users. Increased levels of these biomarkers, thus, could indicate increased oxidative stress related with tampon use. In addition, we found lower levels of PON1P, an antioxidant enzyme known to hydrolyze exogenous organophosphate compounds [29–31], which could indicate decreased ability to combat oxidative stress among tampon users. These increases in oxidative stress biomarkers and decrease in antioxidants may be due to exposure to metals, pesticides or other chemicals present in the tampons.

Tampons may be a source of exposure to chemicals that have been related to oxidative stress and inflammation. DeVito and Schecter [17], for instance, assessed the dioxin content in tampons, and found detectable concentrations of five dioxins in four different brands of tampons with variability in exposure levels across the different brands; however, daily dietary intake of dioxins exceeded daily intake of dioxins from tampons. These findings were consistent with those reported previously [32] in that most of the dioxins found were below the detection limit or estimated detection limits. Importantly, neither of these two studies assumed an absorbed dose via the vaginal route, which may have resulted in under-estimated dioxin exposure from tampons. Experimental evidence confirmed the presence of dioxin in cotton balls [33], with implications for tampons which have yet to be explored. In NHANES 2001–2004, women using tampons had a 6.1 and 4.1% increase in urinary monoethyl phthalate and mono-n-butyl phthalate, metabolites of diethyl phthalate and di-n-butyl phthalate, compared to not using tampons, although the differences were not statistically significant (*n* = 731) [34]. Phthalates are expected to be an important group of chemicals in fragranced tampons, although exposure to tampon-related fragrance chemical in general has not been studied. These studies suggest that tampons may be a source of exposure to various chemicals that are related to oxidative stress and inflammation [35–38].

Our study is the first one, to our knowledge, to investigate tampons as a potential source of exposure to metals and other chemicals that may result in elevated inflammation and oxidative stress. We were able to use data from a well-characterized cohort with detailed information on tampon use and oxidative stress and inflammation biomarkers measured at multiple points during the menstrual cycle. Our findings, nevertheless, should be interpreted in light of our limitations. First, and most importantly, the BioCycle Study was not designed to study tampon use and tampon-related chemical exposures. While the BioCycle Study was designed to study oxidative stress and inflammation, metal exposure was not measured in the same way as the mechanistic biomarkers. The metals were measured from a single whole-blood sample collected approximately 16 days before the beginning of the first menstrual cycle during the study and no additional collection was obtained before the second menstrual cycle. This may not have accurately represented the levels of these metals when the women were using tampons in cycles one and two. However, we believe that tampon use patterns are consistent across cycles, as also shown by the estimated ICC. Given the potentially missed critical exposure window, the fact that other sources of exposure also exist, and that – since the study was not designed for our hypothesis – no other tampon-relevant chemicals were measured (e.g. pesticides), we would expect exposure measurement error to be an important source of bias in our study. However, since there is no reason to believe that any error is related to tampon use, any bias would be towards the null, which may explain our null findings. Tampon use was self-reported; therefore, exposure measurement error is also likely. However, there is no reason to believe that any misclassification in self-reported tampon use is related to the dependent variables included in our analyses, and, thus, any bias is expected to be towards the null. Another limitation of this study is that the sample size is small, limiting our power to detect significant associations. Also, our results may not be generalizable because most of the participants were highly educated. Finally, we cannot exclude the possibility of residual confounding; although we assessed multiple variables as potential confounders, these associations have never before been examined, and we were limited to assess confounding by variables for which we had information. There are substantial fluctuations in the levels of oxidative stress and inflammation biomarkers during the menstrual cycle [39]. Although we cannot exclude the possibility that a variable exists that covaries with these fluctuations and tampon use, we do not believe that this potential source of unmeasured confounding is likely. Rather, the ability to capture these fluctuations with the multiple measurements of these biomarkers during the menstrual cycle is a strength of our study.

## Conclusion

In conclusion, we found suggestive evidence that women who used tampons had increased levels of mercury, and oxidative stress biomarkers during different times of the menstrual cycle, but these increases were not statistically significant. While our study lacked statistical power, exposure to chemicals in tampons and their potential health effects are important public health concerns. The vaginal exposure route has been so far overlooked, although it is an extremely important route because of the rapid absorption that occurs in the vagina and the widespread use of tampons. Indeed, many women could be exposed to chemicals present in tampons as 50–86% of women in the United States report using tampons [1]. Tampon use is a potentially important, yet understudied, source of chemical exposure that could be associated with adverse health. This potentially important public health issue requires additional research efforts, including the chemical assessment of tampons and the conduction of larger and sufficiently-powered biomarker studies of tampon users to assess the importance of tampon use as a chemical exposure pathway.

## Additional file

Additional file 1: **Table S1.** exp(β)^*a*^ of Tampon Use (binary) and 95% Confidence Intervals (95% CIs) for Blood Oxidative Stress and Inflammation Biomarkers for Model 2, restricted to non-smokers. **Table S2.** Percent change^*a*^ in Blood Oxidative Stress and Inflammation Biomarkers for Tampon Use (continuous) and 95% Confidence Intervals (95% CIs) for Model 2. (DOCX 20 kb)
