# Supplementary material for: Tampon use, environmental chemicals and oxidative stress in the BioCycle study
Source: Environ Health. 2019 Feb 11;18:11. doi: 10.1186/s12940-019-0452-z (PMC6371574; doi:10.1186/s12940-019-0452-z)
Supplement: Supplementary file 1 — Table S1. exp(β)a of Tampon Use (binary) and 95% Confidence Intervals (95% CIs) for Blood Oxidative Stress and Inflammation Biomarkers for Model 2, restricted to non-smokers. Table S2. Percent changea in Blood Oxidative Stress and Inflammation Biomarkers for Tampon Use (continuous) and 95% Confidence Intervals (95% CIs) for Model 2. (DOCX 20 kb) [file 12940_2019_452_MOESM1_ESM.docx]

**Supplemental Material**

**Tampon Use, Environmental Chemicals and Oxidative Stress in the BioCycle Study**

Jessica Singh^1^, Sunni L. Mumford^2^, Anna Z. Pollack^3^, Enrique F. Schisterman^2^, Marc G. Weisskopf^4^, Ana Navas-Acien^1^, Marianthi-Anna Kioumourtzoglou^1*^

^1^Department of Environmental Health Sciences, Mailman School of Public Health, Columbia University, New York, New York, USA

^2^Eunice Kennedy Shriver National Institute of Child Health and Human Development, Epidemiology Branch, Rockville, Maryland, USA

^3^Department of Global and Community Health, College of Health and Human Services, George Mason University, Fairfax, Virginia, USA

^4^Department of Environmental Health, Harvard TH Chan School of Public Health, Boston, Massachusetts, USA

**Table of Contents**

Table S1. exp(β) of Tampon Use (binary) and 95% Confidence Intervals (95% CIs) for Blood Oxidative Stress and Inflammation Biomarkers for Model 2, restricted to non-smokers.

Table S2. Percent change in Blood Oxidative Stress and Inflammation Biomarkers for Tampon Use (continuous) and 95% Confidence Intervals (95% CIs) for Model 2.

Table S1. exp(β)*^a^* of Tampon Use (binary) and 95% Confidence Intervals (95% CIs) for Blood Oxidative Stress and Inflammation Biomarkers for Model 2, restricted to non-smokers.

|  | exp(β) (95%CI) | |
| --- | --- | --- |
| TBARS (nmol/mL) |  |  |
| Menses | 0.99 (0.94, 1.05) | |
| Early-follicular Phase | 1.00 (0.95, 1.06) | |
| Menstruating Week | 1.00 (0.95, 1.05) | |
| Cycle | 1.02 (0.98, 1.06) | |
| Cycle except  menstruating week | 1.03 (0.98, 1.08) | |
| PON1A (µmol/min/L) |  |  |
| Menses | 1.04 (1.00, 1.09) | |
| Early-follicular Phase | 1.00 (0.96, 1.04) | |
| Menstruating Week | 1.02 (0.98, 1.06) | |
| Cycle | 1.01 (0.98, 1.04) | |
| Cycle except  menstruating week | 1.00 (0.97, 1.03) | |
| PON1P (µmol/min/L) |  |  |
| Menses | 1.02 (0.90, 1.15) | |
| Early-follicular Phase | 1.01 (0.90, 1.13) | |
| Menstruating Week | 1.00 (0.90, 1.11) | |
| Cycle | 0.95 (0.90, 1.02) | |
| Cycle except  menstruating week | 0.96 (0.90, 1.03) | |
| Isoprostane (pg/mL) |  |  |
| Menses | 1.05 (0.95, 1.15) | |
| Early-follicular Phase | 1.05 (0.96, 1.15) | |
| Menstruating Week | 1.05 (0.96, 1.15) | |
| Cycle | 1.03 (0.96, 1.11) | |
| Cycle except  menstruating week | 1.02 (0.96, 1.09) | |
| CRP (mg/L) |  |  |
| Menses | 1.14 (0.91, 1.43) | |
| Early-follicular Phase | 0.96 (0.77, 1.20) | |
| Menstruating Week | 1.03 (0.84, 1.27) | |
| Cycle | 0.95 (0.77, 1.17) | |
| Cycle except  menstruating week | 0.90 (0.72, 1.11) | |

*^a^*Ratio of the expected geometric mean for those who used tampons over those who did not.

Table S2. Percent change*^a^* in Blood Oxidative Stress and Inflammation Biomarkers for Tampon Use (continuous) and 95% Confidence Intervals (95% CIs) for Model 2.

|  | Percent change (95%CI) | |
| --- | --- | --- |
| TBARS (nmol/mL) |  |  |
| Menses | 0.30 (-0.79, 1.41) | |
| Early-follicular Phase | 0.52 (-0.52, 1.57) | |
| Menstruating Week | 0.34 (-0.62, 1.30) | |
| Cycle | 0.49 (-0.28, 1.21) | |
| Cycle except  menstruating week | 0.55 (-0.21, 1.31) | |
| PON1A (µmol/min/L) |  |  |
| Menses | 0.21 (-0.61, 1.04) | |
| Early-follicular Phase | 0.29 (-0.47, 1.05) | |
| Menstruating Week | 0.18 (-0.48, 0.84) | |
| Cycle | 0.20 (-0.33, 0.73) | |
| Cycle except  menstruating week | 0.23 (-0.36, 0.81) | |
| PON1P (µmol/min/L) |  |  |
| Menses | 1.67 (-0.50, 3.89) | |
| Early-follicular Phase | 0.78 (-1.25, 2.85) | |
| Menstruating Week | 0.92 (-0.93, 2.80) | |
| Cycle | -0.89 (-1.93, 0.34) | |
| Cycle except  menstruating week | -0.87 (-2.06, 0.34) | |
| Isoprostane (pg/mL) |  |  |
| Menses | 0.64 (-1.11, 2.42) | |
| Early-follicular Phase | 1.10 (-0.56, 2.80) | |
| Menstruating Week | 0.94 (-0.72, 2.63) | |
| Cycle | 0.38 (-0.98, 1.75) | |
| Cycle except  menstruating week | 0.19 (-1.03, 1.41) | |
| CRP (mg/L) |  |  |
| Menses | 0.25 (-3.76, 4.42) | |
| Early-follicular Phase | -2.83 (-6.66, 1.16) | |
| Menstruating Week | -1.82 (-5.48, 2.00) | |
| Cycle | -0.88 (-4.62, 3.00) | |
| Cycle except  menstruating week | -1.32 (-5.20, 2.72) | |

*^a^*Percent change of geometric mean for every additional tampon used.
